# Signet ring cell colorectal cancer: genomic insights into a rare subpopulation of colorectal adenocarcinoma

**DOI:** 10.1038/s41416-019-0548-9

**Published:** 2019-08-13

**Authors:** Krittiya Korphaisarn, Van Morris, Jenifer S. Davis, Michael J. Overman, David R. Fogelman, Bryan K. Kee, Arvind Dasari, Kanwal P. S. Raghav, Imad Shureiqi, Metha Trupti, Robert A. Wolff, Cathy Eng, David G. Menter, Stanley Hamilton, Scott Kopetz

**Affiliations:** 1 Department of Gastrointestinal Medical Oncology, The University of Texas MD Anderson Cancer Center, Houston, TX USA; 2 Division of Medical Oncology, Department of Medicine, Faculty of Medicine Siriraj Hospital, Mahidol University, Bangkok, Thailand; 3 Department of Epidemiology, The University of Texas MD Anderson Cancer Center, Houston, TX USA; 4 Department of Pathology, The University of Texas MD Anderson Cancer Center, Houston, TX USA

**Keywords:** Colorectal cancer, Cancer genomics

## Abstract

**Background:**

Signet ring cell carcinoma (SRCC) is a rare subtype of colorectal cancer (CRC). The aim of this study was to characterise the genomic alterations and outcomes of SRCC.

**Methods:**

Medical records of metastatic CRC (mCRC) patients whose tumours were evaluated by NGS analysis were reviewed. SC-mCRC were classified into two groups: SRCC (>50% signet ring cells) and adenocarcinoma (AC) with SC component (≤50% signet ring cells).

**Results:**

Six hundred and sixty-five mCRC patients were included. Of the 93 mCRC cases with SC features, 63 had slides for review. Of those 63 cases, 35 were confirmed SRCC, and 28 were AC with SC component. Compared with AC group, *KRAS and PIK3CA* mutations (mts) were found in only 11% (OR: 0.13) and 3% (OR: 0.15) of SRCC cases, respectively. In contrast to the 44% rate of *APC* mts in AC group, only 3% of SRCC patients had *APC* mts (OR = 0.04).

**Conclusions:**

SRCC has distinct molecular features, including low rates of *KRAS*, *PIK3CA* and *APC* mts. Further study to identify activation pathways and potential therapeutic targets are needed.

## Background

Colorectal cancer (CRC) is the third most commonly diagnosed cancer, and the fourth leading cause of cancer death in the world.^1^ CRC has a variety of histologies, including adenocarcinoma (AC), and rare histologies, such as adenosquamous carcinomas, squamous cell carcinomas, neuroendocrine carcinomas, spindle cell carcinomas and undifferentiated carcinomas.^2^ Signet ring cell colorectal cancer (SRCC) is a rare subtype of colorectal adenocarcinoma that accounts for 1–2.4% of all CRC.^3,4^ The histology of SRCC is distinguished from typical adenocarcinoma by an excess amount of intracellular mucin that displaces the nucleus, which results in the formation of signet ring cell (SC) features. SRCC is defined by a greater than 50% presence of signet ring cells, while cases with less than or equal to 50% presence of signet ring cells are noted to have signet ring features without a formal SRCC designation.^5^ In the previous studies, SRCC has been associated with younger age, advanced tumour stage at presentation and lymph node metastasis,^2,6–8^ and SRCC has significantly poorer prognosis compared to that of adenocarcinomas.^6,9–14^ Several studies have suggested a higher rate of microsatellite instability and *BRAF* gene mutation in SRCC.^7,15–17^ However, the sample sizes in those studies were generally small, and most evaluated only single gene mutation. A more extensive clinical and molecular characterisation of this subset is needed. Accordingly, the aim of this study was to characterise the genomic alterations, clinicopathological characteristics and outcomes of SRCC.

## Methods

This single centre, retrospective cohort study evaluated patients with metastatic CRC (mCRC) who were enrolled in the Assessment of Targeted Therapies Against Colorectal Cancer (ATTACC) program at The University of Texas MD Anderson Cancer Center (UTMDACC) between 13 February 2009 and 18 November 2015, as described previously.^18^ Additional mCRC cases having signet ring cell features between 1 March 1994 and 31 August 2015 were extracted from the tumour registry at UTMDACC. The protocol for this study was approved by The University of Texas MD Anderson Cancer Center Institutional Review Board. All patients provided written informed consent prior to sequencing of their tumours according to the institutional guideline. The primary objective of this study was to determine the molecular characteristics of SRCC. The secondary objectives were to identify significant associations between SRCC and various clinicopathologic characteristics, and to evaluate their prognostic impact on overall survival (OS).

### Pathologic evaluation

Slides from patients whose pathology report documented the presence of signet ring cell histology were obtained and reviewed to confirm the percentage of signet ring cells. Haematoxylin and eosin-stained slides of the tumours were reviewed by an experienced gastrointestinal pathologist (SH). Tumours were classified according to the proportion of signet ring cells, with ≥50% defining SRCC, and ≤50% defining AC with SC component.

### Clinical characteristics

As described previously,^18^ demographic and clinical information, including age, gender, race, primary tumour site, date of diagnosis with stage IV disease, date of last follow-up and date of death, were collected from a review of patient medical records. Right-sided colon cancer was defined as cancer in the region from the cecum to the splenic flexure, while left-sided colon cancer was defined as cancer in the region from the descending colon through the sigmoid colon, and the rectum was considered a separate site. Staging was performed using the American Joint Committee on Cancer/Union for International Cancer Control TMN staging system (version 7, 2010). OS was defined as the interval between the date of diagnosis of metastatic disease and the date of death from any cause. Patients alive at the time of analysis were censored at their last known follow-up date

### Molecular characterisation

As described previously,^18^ DNA was extracted from paraffin-embedded formalin-fixed tumour tissue. Samples were evaluated using a next-generation sequencing platform with 46- or 50-gene panels for the detection of frequently reported point mutations in human malignancies. Complete details of exon and codon coverage in all genes were previously reported.^19^ DNA testing was performed in a Clinical Laboratory Improvement Amendments-certified molecular diagnostics laboratory that determined the effective lower limit of detection (analytical sensitivity) for single nucleotide variations to be in the range of 5% (one mutant allele in the background of nineteen wild-type alleles) to 10% (one mutant allele in the background of nine wild-type alleles). Details relating to the codons and exons tested are shown in Supplementary Table 1.

### Determine of MMR status

MMR status was determined by analysis of MMR protein expression by immunohistochemistry (IHC) or microsatellite instability (MSI) testing. Deficient mismatch repair (dMMR) was defined as the presence of either high-level MSI (MSI-H) or loss of MMR protein expression. Proficient mismatch repair (pMMR) was defined as the presence of either microsatellite stable (MSS)/low-level MSI (MSI-L) or the presence of normal MMR protein expression. Complete details of IHC analysis of MMR expression and microsatellite instability (MSI) testing were previously published.^18^

### CpG Island Methylation Phenotype (CIMP) panel methylation analysis

As described previously,^20^ DNA extracted from formalin-fixed, paraffin-embedded tissue was treated with bisulfite to convert unmethylated cytosine to uracil. PCR amplification of unmethylated and methylated MINT1, MINT2 and MINT31 loci, and promoter sequences of p14, p16 and hMLH1 genes was performed, and methylation status was assessed by pyrosequencing. The tumour was considered CIMP High if at least 40% of the markers tested show methylation, and CIMP Low if <40% of the tested markers show methylation.

### Statistical analysis

Patient characteristics were compared using descriptive statistics. Pearson’s Chi-square test or Fisher’s exact test was applied to evaluate associations between SRCC and clinicopathological variables, and binary logistic regression was used to calculate odds ratios. Survival was analysed using the Kaplan–Meier method, and comparisons between groups were made using the log-rank test. Cox proportional hazard models were used to estimate the combined influence of clinical and pathologic feature on survival. Median follow-up time was calculated using the reverse Kaplan–Meier method. A two-sided level of significance of 0.05 was applied for all statistical tests. Calculations were performed using SPSS Statistics version 23.0 software (IBM Corp., Armonk, NY, USA).

## Results

Six hundred and sixty-five mCRC patients with NGS molecular data were included. Of the 93 mCRC cases with SC features, 63 had available slides for confirmatory review. Of those 63 cases, 35 were confirmed SRCC, and 28 were considered AC with SC component (Supplementary Fig. 1).

The frequency of confirmed SRCC and AC with SC components in the entire cohort was 5.5% (35/635) and 4.4% (28/635), respectively. However, if analysis only the data form ATTACC database, 1.3% (8/604) and 2.3% (14/604) were classified as SRCC and AC with SC components, respectively. The median age was 55 years (range: 15–85), the ratio of males to females was 1.29, and the majority of patients were Caucasian. Patient and tumour characteristics are shown in Table 1.

**Table 1:** Demographic and clinical characteristics of the study population, n (%)

Variable	*n*	%
No. of patients	665	100
Age (years), median (range)	55 (15–85)	
Gender		
Female	291	43.8
Male	374	56.2
Age		
15–39	70	10.5
40–49	153	23.0
≥50	442	66.5
Site		
Right-sided	243	36.5
Left-sided	274	41.2
Rectum	142	21.4
No data	6	0.9
Race		
Asian	34	5.1
Black	60	9.0
Hispanic	61	9.2
White	503	75.6
No data	7	1.1
Histology		
Adenocarcinama (AC)	572	86.0
Signet ring cell features (*n* = 93)		
Confirmed SRCC	35	5.3
AC with SC	28	4.2
No slide reviewed	30	4.5
Differentiated		
Well	1	0.2
Moderately	449	67.5
Poorly	209	31.4
Not available	6	0.9
Liver metastasis		
No	201	30.2
Yes	464	69.8
Lung metastasis		
No	262	39.4
Yes	403	60.6
Peritoneal metastasis		
No	400	60.2
Yes	265	39.8

*SRCC* signet ring cell colorectal cancer, *AC with SC* adenocarcinoma with signet ring cell component, *AC* adenocarcinoma

### Clinicopathologic features

Compared with the AC group, SRCC was significantly more commonly found in patients with right-sided tumour (odds ratio [OR]: 3.4, *p* = 0.001), poorly differentiated tumour (*p* < 0.001) or peritoneal metastasis (OR: 9.8, *p* < 0.001). In contrast, SRCC was significantly less frequently found in cases with liver metastasis (OR: 0.1, *p* < 0.001) or lung metastasis (OR: 0.1, *p* < 0.001). Patients with SRCC were 2.4-fold more likely to be diagnosed before the age of 40 (*p* = 0.04) (Table 2).

**Table 2:** Clinical characteristics of study population by histology (only reviewed slide cases; *n* = 635)

Variable	Histology						*p*-value*
	SRCC	%	AC with SC	%	AC	%	
Gender							
Female	18	51.4	14	50.0	245	42.8	0.32
Male	17	48.6	14	50.0	327	57.2	
Age							
15–39	7	20.0	2	7.1	53	9.3	0.10
40–49	9	25.7	6	21.4	136	23.8	
≥50	19	54.3	20	71.4	383	67.0	
Site (*n* = 602)							
Right-sided	22	62.9	12	42.9	189	33.3	**0.001**
Left-sided	13	37.1	16	57.1	378	66.7	
Race (*n* = 602)							
Asian	1	2.9	0	0.0	32	5.6	0.43
Black	4	11.8	2	7.1	53	9.3	
Hispanic	2	5.9	2	7.1	53	9.3	
White	27	79.4	24	85.7	430	75.7	
Differentiated (*n* = 601)							
Well-moderately	0	0.0	0	0.0	449	79.3	<**0.001**
Poorly	35	100	28	100	117	20.7	
Liver metastasis							
No	29	82.9	`15	53.6	132	23.1	<**0.001**
Yes	6	17.1	13	46.4	440	76.9	
Lung metastasis							
No	29	82.9	22	78.6	184	32.2	<**0.001**
Yes	6	17.1	6	21.4	388	67.8	
Peritoneal metastasis							
No	6	17.1	7	25.0	383	67.0	<**0.001**
Yes	29	82.9	21	75.0	189	33.0	
MMR status (*n* = 472)							
pMMR	29	87.9	19	90.5	404	95.1	0.10
dMMR	4	12.1	2	9.5	21	4.9	
CIMP (*n* = 215)							
CIMP-L/neg	2	66.7	5	71.4	175	75.1	–^a^
CIMP-H	1	33.3	2	28.6	58	24.9	

The Bold values are statistically significant

A *p*-value < 0.05 indicates statistical significance

*SRCC* signet ring cell colorectal cancer, *AC with SC* adenocarcinoma with signet ring cell component, *AC* adenocarcinoma

*p-value for SRCC compared with AC

^a^No statistical analysis due to small sample size

### Molecular characteristics

**Fig. 1:**
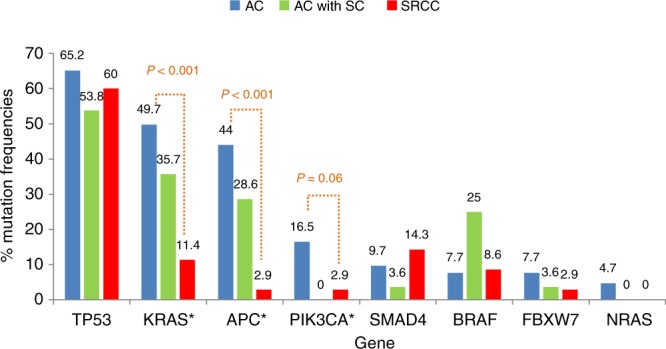
AC molecular characteristics of adenocarcinoma, AC with SC adenocarcinoma with signet ring cell component and SRCC signet ring cell colorectal cancer

Two hundred and six cases were tested with the 46-gene panel while 429 cases were tested with the 50-gene panel. Details relating to gene mutation frequencies in the SRCC, AC with SC component, and AC groups are shown in Fig. 1. Compared with the AC group, SRCC was commonly found with *KRAS* wild-type (wt) (OR: 7.7, 95% CI: 2.7–22.0; *p* < 0.001), *APC* wt (OR: 26.8, 95% CI: 3.6–196.9; *p* = 0.001) and *PIK3CA* wt (OR: 6.7, 95% CI: 0.9–49.9; *p* = 0.06). No significant association was observed between SRCC and MSI, *NRAS*, *BRAF*, *SMAD4*, *TP53* or *FBXW7* status. No difference in gene mutation detection was noted between the two gene panels except in *APC* gene (46.6% in 50-gene panel vs 29.1% in 46-gene-panel, *p* < 0.001) (Supplementary Table 2). AC with SC component trended to have an intermediate prevalence of mutation in *KRAS, APC and FBXW7* between SRCC and AC group (Fig. 1).

### Patient outcomes

**Fig. 2:**
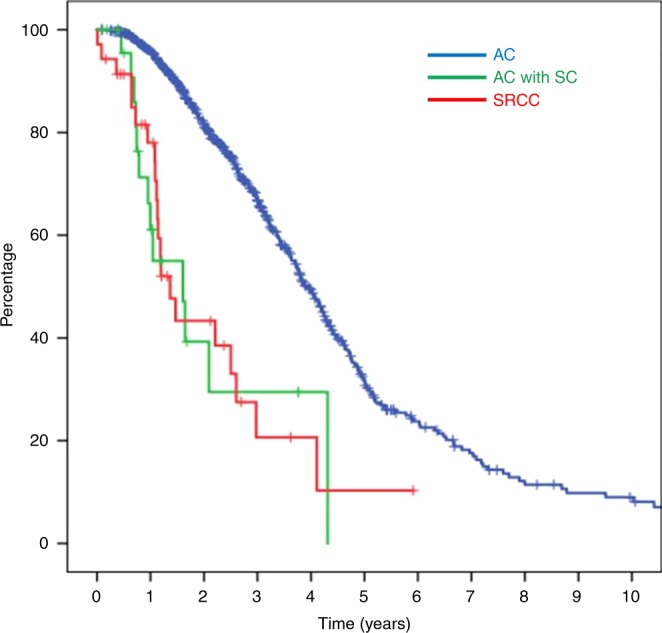
Kaplan–Meier survival curve of CRC patients according to signet ring cell histology. Patients with SRCC had significantly worse OS (median overall survival [OS]: 16.4 mo, 95% confidence interval [CI]: 11.3–21.5) than patients with AC (median OS: 47.2 mo, 95% CI: 43.6–50.9) (*p* < 0.001)

The median follow-up time was 27 months. Univariate analysis by Kaplan–Meier survival analysis and log-rank test was performed using several factors, including age, primary tumour site, histological grade, histological type, *KRAS, BRAF, PIK3CA* and MSI status. Factors found to be significantly associated with worse OS were age 15–39 years (*p* = 0.002), right-sided tumour (*p* < 0.001), poor differentiation (*p* < 0.001), signet ring cell feature (*p* < 0.001), *KRAS* mutation (*p* = 0.013), *BRAF* mutation (*p* < 0.001) and pMMR (*p* = 0.016). Patients with SRCC tumours had significantly worse OS than patients with AC-mCRC (median OS: 16.4 months, 95% CI: 11.3–21.5 *vs*. median OS 47.2 months, 95% CI: 43.6-50.9, respectively; *p* < 0.001) (Table 3, Fig. 2).

**Table 3:** Survival analyses^a^

Variables	Univariate analysis				Multivariate analysis			
	*n*	Median survival (mo)	95% CI	*p*-value	*n*	HR	95% CI	*p*-value
Age (*n* = 662)								
15–39 years	70	36.8	27.0–46.6	**0.002**	52	1.57	0.98–2.53	0.060
40–49 years	152	41.2	32.4–49.9		123	1.34	0.97–1.86	0.077
≥50 years	440	46.2	42.2–50.2		293	Ref.		
Site (*n* = 656)								
Right-sided	242	35.7	30.6–40.8	<**0.001**	163	1.28	0.94–1.74	0.12
Left-sided	414	48.8	44.9–52.7		305	Ref.		
Differentiated (*n* = 656)								
Well-moderately	450	48.5	44.5–52.4	<**0.001**	331	Ref.		
Poorly	206	31.6	25.0–38.1		137	1.35	0.96–1.90	0.088
*KRAS* (*n* = 661)								
Wild-type	358	48.2	43.1–53.4	**0.013**	259	Ref.		
Mutant	303	40.7	35.1–46.4		209	1.51	1.17–1.95	**0.002**
*BRAF* (*n* = 662)								
Wild-type	602	45.7	42.1–49.3	<**0.001**	425	Ref.		
Mutant	60	35.9	15.8–56.0		43	1.86	1.20–2.89	**0.005**
*PIK3CA* (*n* = 658)								
Wild-type	560	45.6	41.6–49.6	0.111				
Mutant	98	42.4	33.6–51.1					
MMR status (*n* = 498)								
Proficient	470	44.8	40.3–49.4	**0.016**	442	Ref.	1.20–3.90	**0.010**
Deficient	28	35.9	8.5–63.4		26	2.17		
Histology (*n* = 633)								
AC	572	47.2	43.6–50.9	<**0.001**	416	Ref.		
AC with SC	26	19.3	10.7–27.8		19	2.63	1.30–5.33	**0.007**
SRCC	35	16.4	11.3–21.5		33	3.11	1.73–5.60	<**0.001**

The Bold values are statistically significant

A *p*-value < 0.05 indicates statistical significance

*CI* confidence interval, *HR* hazard ratio, *Ref.* reference, *SRCC* signet ring cell colorectal cancer, *AC with SC* adenocarcinoma with signet ring cell component, *AC* adenocarcinoma

^a^Only conducted among patients with available data

Multivariate Cox proportional hazard regression analysis revealed *KRAS* mutation (HR: 1.51, 95% CI: 1.17–1.95; *p* = 0.002), *BRAF* mutation (HR: 1.86, 95% CI: 1.20–2.89; *p* = 0.005) and pMMR (HR: 2.17, 95% CI: 1.20–3.90; *p* = 0.010) as independent predictors of worse outcome. After adjusting for all of these features, SRCC remained a significant prognostic factor for poor OS (HR: 3.11, 95% CI: 1.73–5.6; *p* < 0.001) (Table 3).

## Discussion

This study identified the molecular characteristics of SRCC, which is a rare subtype of colorectal adenocarcinoma. We demonstrated associations between SRCC and *KRASwt*, *PIK3CAwt* and *APCwt*. This study also confirmed that SRCC is associated with young onset, right-sided tumour, peritoneal metastasis and poor outcome.

Adenocarcinoma is the most common histologic type of CRC, accounting for more than 90% of CRC cases. Mucinous adenocarcinoma (MAC) and SRCC are less commonly observed subtypes, with MAC accounting for 10% of cases, and SRCC accounting for 1% of cases.^8,9,21–23^ SRCC is defined by the presence of >50% of tumour cells with intracytoplasmic mucin, whereas MAC is defined as carcinoma with >50% of the tumour volume showing extracellular mucin.^24^ Due to the rarity of this subtype and the difficulties associated with characterising it molecularly, genomic alteration data in SRCC are scarce. A summary of previously reported molecular alterations in SRCC are provided in Table 4.

**Table 4:** Clinical studies that investigating molecular features, MSI-H and CIMP-H in signet ring cell colorectal cancer

Study	n	Stage	Histology	*KRAS* (*n*, %)	*NRAS* (*n*, %)	*BRAF* (*n*, %)	*PIK3CA* (*n*, %)	*FBXW7* (*n*, %)	*APC* (*n*, %)	*TP53* (*n*, %)	*SMAD4* (*n*, %)	MSI-H (*n*, %)	CIMP-H (*n*, %)
Present study	35	IV	Confirmed ≥ 50% SC	4/35 (11.4%)	0/35 (0.0%)	3/35 (8.6%)	1/35 (2.9%)	1/35 (2.9%)	1/35 (2.9%)	21/35 (60.0%)	5/35 (14.3%)	4/33 (12.1%)	1/3 (33.3%)
Kawabata Y, 1999^24^	10	II–IV	Not confirmed	1/9 (11.0%)	–	–	–	–	–	–	–	3/10 (30.0%)	–
Kakar S, 2005^4^	45	I–IV	Confirmed ≥ 50% SC	–	–	–	–	–	–	–	–	12/45 (26.7%)	–
Ogino S, 2006^25^	9	NA	Confirmed ≥ 50% SC	0/8 (0.0%)	–	2/9 (22.0%)	–	–	–	–	–	2/8 (25.0%)	–
Kakar S, 2012^17^	33	I–IV	Confirmed ≥ 50% SC	16/30 (53.3%)	–	9/27 (33.3%)	–	–	–	–	–	8/33 (24.2%)	16/33 (48.5%)
Hartman DJ, 2013^16^	53	I–IV	Confirmed ≥ 50% SC	–	–	16/50 (32.0%)	–	–	–	–	–	23/53 (43.4%)	–
Inamura K, 2015^15^	20	I–IV	Confirmed ≥ 50% SC	1/17 (5.9%)	–	6/17 (35.3%)	1/16 (6.2%)	–	–	–	–	5/17 (29.4%)	5/17 (29.4%)
Nitsche U, 2016^12^	160	I–IV	Not confirmed	–	–	–	–	–	–	–	–	1/5 (20.0%)	–
Wei Q, 2016^7^	39	I–IV	Confirmed ≥ 50% SC	5/33 (15.2%)	–	1/33 (3.0%)	–	–	–	–	–	–	–
Yalcin S, 2017^28^	9	II–IV	Confirmed ≥ 50% SC	–	–	4/9 (36.4%)	–	–	–	–	–	–	–
Nam JY, 2018^27^	5	NA	Confirmed > 50% SC	2/5 (40.0%)	0/5 (0.0%)	–	0/5 (0.0%)	1/5 (20.0%)	1/5 (20.0%)	2/5 (40.0%)	1/5 (20.0%)	–	–

Our study identified a low rate of *KRASmt* (11.4%) in SRCC, which is comparable to the rates reported in many, but not all previous studies of SRCC.^7,17,25–28^ However, and importantly, the larger sample size in our study, our attention to confirmation of the histologic diagnosis, and use of NGS in a clinical lab should influence increased confidence in our results. The rate of MSI-H in this study was lower than previously reported in SRCC.^4,12,15–17,25,26,29^ This is likely due to the fact that our population was limited to stage IV disease, which normally has lower rate MSI-H compared to earlier stage disease.

Previous studies by Kakar S et al.^17^ and Inamura K et al.^15^ found *BRAF V600E* mutation and CIMP-positive status to be more common in SRCC, and proposed that SRCC might be related to the serrated pathway, based on the increased prevalence of *BRAF V600E*mt and CIIMP-positive status in a majority of serrated polyps.^30^ However, in metastatic disease, the findings of our study suggest that this association may not be as clear, and suggests the involvement of alternate carcinogenesis pathways.

Phosphatidylinositol-4,5-biphosphonate 3-kionase (*PIK3CA*) mutations have been reported in 10–20% of all CRC.^31^ However, no detectable *PIK3CA* mutation was found in SRCC in this study, which is consistent with the findings of Inamura et al. who reported a prevalence of only 6.2% (1/16 cases) in SRCC.^15^ Adenomatous polyposis coli (*APC)* mutations are the most commonly acquired mutation in sporadic colon cancer, and are considered the initial genetic alteration in CRC tumorigenesis.^32^ Interestingly, our study found *APCmt* in only 3% of SRCC compared to 44% in AC group.

In the present study, we also reported the gene mutation frequencies in AC with SC component mCRC. Interestingly, the frequencies in most of the genes were similar to those observed in non-SC mCRC, while the frequencies in the other genes were varied between those observed in SRCC and those observed in non-SC mCRC (Fig. 2). This could be influenced by mixed component of SRCC and conventional adenocarcinoma during the tissue selection process. It is, therefore, strongly encouraged to define the patient as either SRCC or AC with signet ring cell component.

When compared with conventional adenocarcinoma, SRCC has distinct clinicopathological characteristics. SRCC was reported to be predominately observed in younger onset group (especially in patients aged less than 40 years), and to be associated with right-sided tumour, advanced stage at presentation, and poor prognosis.^8–10,12,33–35^ The reported 5-year survival in the literature was 28.6–33%,^17,21^ but only 4.5% in stage IV disease.^21^ Peritoneal carcinomatosis is the most common site of metastasis.^3^ In our study, we found SRCC to be more commonly found in younger aged patients (especially age 15–39 years), right-sided tumour, poorly differentiated histology, more frequently with peritoneal metastasis over lung or liver metastasis and significantly inferior OS compared with non-SRCC tumours. We also found the worse prognosis of SC histology to be similar between SRCC and AC with SC component. We, therefore, conclude that any presence of signet ring cells of any proportion in CRC leads to poorer clinical outcomes.

This study has some mentionable limitations. First and consistent with the retrospective nature of this study, some patient data may have been missing or incomplete. Second, the data included in this study was from a single centre. Third, the sequencing panels that were used were limited to hotspot regions of several tumour suppressor genes. Therefore, the presence of other mutations outside of these regions cannot be excluded. Finally, the small number of included samples due to the rarity of SRCC may have given our study insufficient statistical power to identify all significant associations and differences. However, to the best of our knowledge, this is the largest study to evaluate the molecular profiles of SRCC. Further study to determine the key mechanism of tumour development is needed to improve treatments and patient outcomes.

## Conclusion

Colorectal SRCC has distinct molecular features, including low rates of *KRAS*, *PIK3CA* and *APC* mutations. Its unique clinical features and association with early age of disease onset necessitate further study to identify activation pathways and potential therapeutic targets.

## Supplementary information

Supplementary Figure and Tables

## Data Availability

The datasets used and/or analysed during the current study are available from the corresponding author on reasonable request.
