# Supplementary material for: Signet ring cell colorectal cancer: genomic insights into a rare subpopulation of colorectal adenocarcinoma
Source: Br J Cancer. 2019 Aug 13;121(6):505–10. doi: 10.1038/s41416-019-0548-9 (PMC6738104; doi:10.1038/s41416-019-0548-9)
Supplement: Supplementary file 1 — Supplementary Figure and Tables [file 41416_2019_548_MOESM1_ESM.docx]

**Supplementary Figure 1**. CONSORT flow diagram showing the enrollment and allocation of the study population. There were 604 patients who were enrolled into ATTACC program, and 61 patients with NGS data and histology with signet ring cell features. Ninety-three cases in total were noted to have signet ring cell feature. Of those, 35 patients were confirmed SRCC, 38 patients were AC with SC component, and 30 cases had no slides for review.


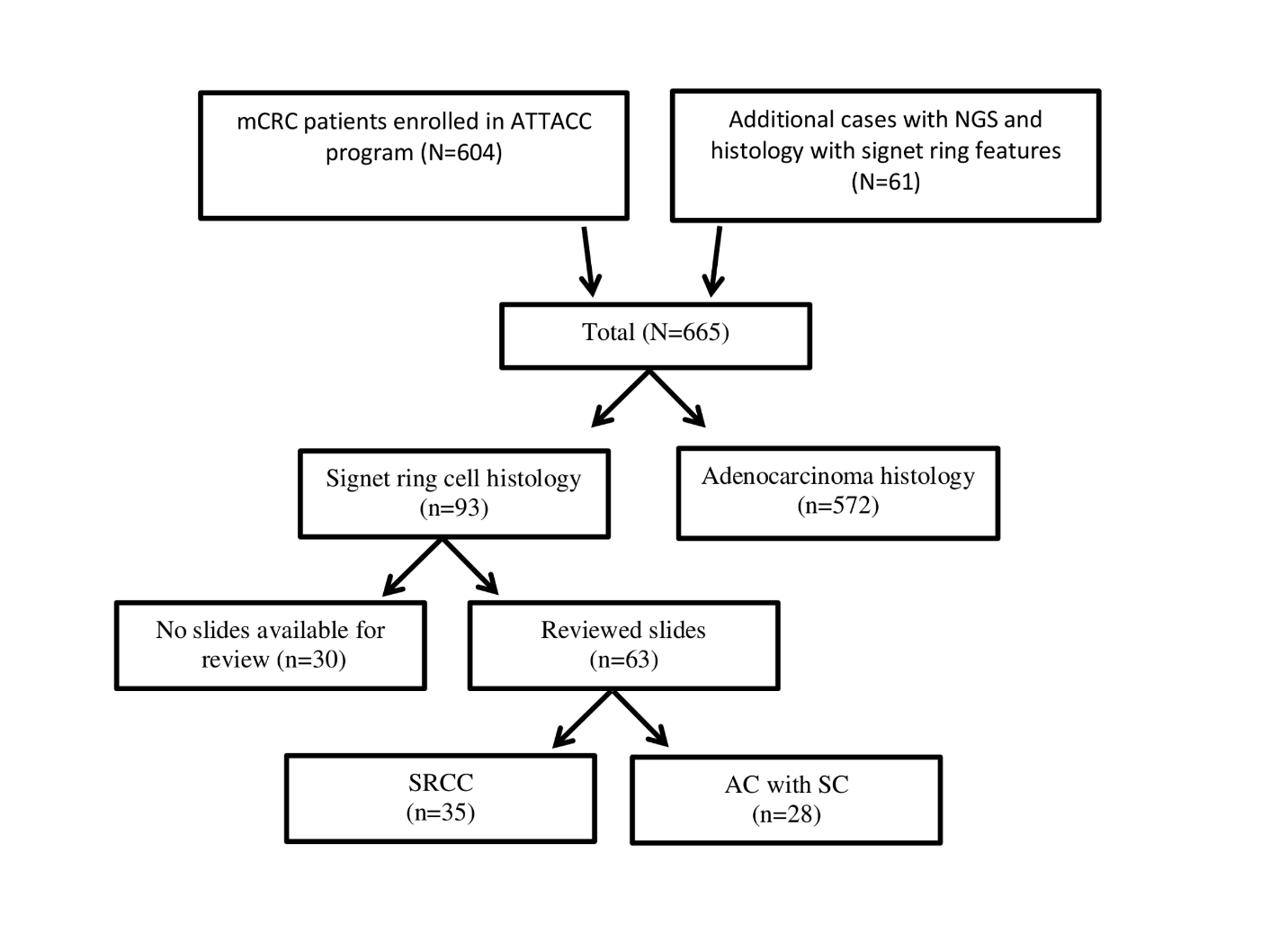


**Supplementary Table 1.** Details of the codon and exon coverage in reported genes

| **Genes** | **CMS 46**  **Exon (codon)** | **CMS 50**  **Exon (codon)** |
| --- | --- | --- |
| ***KRAS*** | 2(5-28), 3(40-67), 4(136-150) | 2-3(5-66), 4(114-150) |
| ***NRAS*** | 2(6-22), 3(53-69) | 2(3-31), 3(43-69), 4(124-150) |
| ***BRAF*** | 11(439-471), 15(581-605) | 11(439-473), 15(581-611) |
| ***PIK3CA*** | 2(77-98), 5(328-351), 8(418-422),  10(533-551), 14(688-716), 21(1065-1069) | 2(54-90), 2(116-118), 5(316-351),  7-8(390-422), 8(449-468), 10(522-549), 14(677-720), 19(898-924), 21(1017-1051), 21(1065-1069) |
| ***FBXW7*** | 5(264-279), 8(381-400), 9(450-472),  10(478-506), 11(566-583) | 5(264-287), 8(378-403), 9(434-473),  10(478-509), 11(567-594) |
| ***APC*** | 16(865-886), 16(1105-1112), 16(1289-1322), 16(1349-1382), 16(1487-1509),  16(1549-1564), | 16(860-891), 16(1089-1125), 16(1284-1326), 16(1342-1384), 16(1426-1471), 16(1483-1524), 16(1543-1582) |
| ***TP53*** | 2(1-18), 4(81-114), 5(126-135), 5(149-181), 6(187-223), 7(230-253), 8(269-306),  10(332-344) | 2(1-20), 4(68-113), 5(126-138),  5-6(149-223), 7(225-258), 8(263-307), 10(332-367) |
| ***SMAD4*** | 3(109-128), 5(167-184), 6(228-247), 8(304-319), 9(330-363), 10(385-404), 11(444-472), 12(497-526) | 3(98-136), 4(142-146), 5(165-202), 6(242-263), 8(307-319), 9(326-365), 10(384-424),  11(443-474), 12(494-532) |

**Supplementary Table 2.** Gene mutation frequency in 46-, 50- gene panels

| Genes | 50-gene panel | | 46-gene panel | | P value |
| --- | --- | --- | --- | --- | --- |
|  | wt | mt | wt | mt |  |
| *TP53* | 143 | 280 | 75 | 115 | 1.18 |
| *KRAS* | 233 | 195 | 103 | 103 | 0.29 |
| ***APC*** | **228** | **199 (46.6%)** | **141** | **58 (29.1%)** | **<0.001** |
| *PIK3CA* | 363 | 63 | 273 | 32 | 0.79 |
| *SMAD4* | 385 | 43 | 185 | 18 | 0.64 |
| *BRAF* | 395 | 34 | 186 | 20 | 0.45 |
| *FBXW7* | 397 | 32 | 192 | 14 | 0.76 |
| *NRAS* | 413 | 16 | 195 | 11 | 0.35 |
